# Effect of the 2014 Clinical and Laboratory Standards Institute urine-specific breakpoints on cefazolin susceptibility rates at a community teaching hospital

**DOI:** 10.1186/s12941-017-0217-x

**Published:** 2017-05-30

**Authors:** Daniel B. Chastain, Ijang Ngando, Christopher M. Bland, Carlos Franco-Paredes, W. Anthony Hawkins

**Affiliations:** 1 University of Georgia College of Pharmacy, 1000 Jefferson Street, Albany, GA 31701 USA; 2 Phoebe Putney Memorial Hospital, Albany, GA 31701 USA; 3 Beaufort Memorial Hospital, Beaufort, SC 29902 USA; 4 University of Georgia College of Pharmacy, Savannah, GA 31405 USA; 5 Hospital Infantil de Mexico, Federico Gomez, Mexico City, Mexico; 6 Medical College of Georgia at Augusta University, Albany, GA 31701 USA

**Keywords:** Antibiotic susceptibility, Microbiology, Urinary tract infections (UTI)

## Abstract

**Background:**

Enterobacteriaceae, which include *Escherichia coli, Klebsiella pneumoniae,* and *Proteus mirabilis*, are identified as the infectious etiology in the majority of urinary tract infections (UTIs) in community hospitals across the United States. The minimum inhibitory concentration (MIC) is a useful tool when choosing an appropriate antibacterial agent. Recent changes to the 2014 Clinical and Laboratory Standards Institute (CLSI) guidelines included reporting a urine-specific cefazolin breakpoint for enterobacteriaceae (susceptible ≤16 mcg/mL). The purpose of this study was to determine the clinical and financial impact of implementing the 2014 CLSI urine-specific breakpoints for cefazolin in a community-based teaching hospital in the Southern U.S.A.

**Methods:**

A retrospective review of patients hospitalized from January 1, 2010 through October 1, 2014 was performed. Patients that met inclusion criteria had a documented initial clinical isolate of *E. coli, K. pneumoniae*, or *P. mirabilis* from urine cultures during each year. Descriptive statistics and two-proportion test of hypothesis were used in the analysis to compare susceptibility rates before and after implementation of the updated CLSI breakpoints for cefazolin.

**Results:**

A total of 190 clinical isolates from patients were included in the study. *E. coli* was the most common organism isolated (63.7%), followed by *K. pneumoniae* (22.1%), and *P. mirabilis* (14.2%). 86% of the included isolates were susceptible to cefazolin using the 2010 breakpoints. Implementation of the 2014 breakpoints did not significantly impact susceptibility results for *E. coli, K. pneumoniae*, or *P. mirabilis.*

**Conclusion:**

Modification of breakpoints did not significantly impact susceptibility rates of cefazolin. Substituting cefazolin may decrease the overall drug cost by 77.5%. More data is needed to correlate in vitro findings with clinical outcomes using cefazolin for UTIs.

## Background

Urinary tract infections (UTIs) account for over 7 million healthcare provider visits annually as well as 1 million emergency visits which result in 100,000 hospitalizations [1, 2]. The majority of microorganisms that cause UTIs in community hospitals across the United States are enterobacteriaceae, which include *Escherichia coli, Klebsiella pneumoniae,* and *Proteus mirabilis* [1, 3]. UTIs are diagnosed by assessing patient symptoms (e.g. dysuria, increased urinary frequency) in combination with urinalysis. A urine culture is typically used to identify the responsible pathogen(s). Following organism identification, determination of antimicrobial susceptibility is crucial in determining appropriate targeted antimicrobial therapy [3].

Minimum inhibitory concentrations (MICs) provide quantitative information about the antibacterial agents’ in vitro activity against the isolated organism [4]. The MIC value must therefore be interpreted in combination with clinical parameters, including pharmacokinetic (PK) and pharmacodynamic (PD) properties of the drug and the site of infection. Certain antibacterial agents, such as β-lactams and fluoroquinolones (FQs), achieve higher urinary concentrations than others. However, clinically some β-lactams have been shown to not be as effective as FQs despite adequate urinary concentrations [5]. Despite these discordant outcomes, most studies have shown that urine concentrations of antimicrobials are better predictors of treatment success than are serum concentrations.

The Clinical and Laboratory Standards Institute (CLSI) publishes guidelines for conducting and interpreting in vitro antibiotic susceptibility testing (AST) [6]. AST is an indispensable component of the microbiology laboratory and is often used to aid clinicians in identifying susceptible and resistant antibacterial agents [7]. In January 2010, CLSI updated the MICs for cefazolin when enterobacteriaceae are isolated from blood cultures [6] (Table 1). These changes were to account for the mechanisms of drug-resistance observed in documented treatment failures in patients with enterobacteriaceae bacteremia treated with cephalosporins. The primary concern was that the new MICs for cefazolin (susceptible [≤1 mcg/mL], intermediate [2 mcg/mL], and resistant [≥4 mcg/mL]) could eventually eliminate its use in the treatment of organisms lacking the expression of AmpC β-lactamases [3].

**Table 1:** Comparison of susceptibility breakpoints for cefazolin

Cefazolin	Susceptible (mcg/mL)	Intermediate (mcg/mL)	Resistant (mcg/mL)
CLSI guidelines: revisions to serum breakpoints			
Pre-2010	≤8	16	≥32
2010–2014	≤1	2	≥4
CLSI guidelines: addition of urine-specific breakpoint			
2014	≤16	–	≥32

The 2014 CLSI guidelines retained the current MICs for cefazolin against enterobacteriaceae, but specified that susceptible results were based on a dosing regimen of 2 grams intravenously every 8 h and is specific for non-urine isolates [8]. One of the major changes included in these guidelines involves the reporting of a urine-specific cefazolin MIC for enterobacteriaceae (susceptible ≤16 mcg/mL). Furthermore, for uncomplicated UTIs caused by *E. coli*, *K. pneumoniae*, and *P. mirabilis*, cefazolin may be used as a surrogate to predict susceptibility to oral cephalosporins such as cephalexin and cefpodoxime. The purpose of this study was to determine the potential impact of implementing the 2014 CLSI urine-specific MICs for cefazolin.

## Methods

This was a single-center retrospective cohort study of hospitalized patients at a 671-bed community-based teaching hospital in Southwest Georgia, U.S.A. The study was approved by the institutional review boards of the hospital and affiliated university. Microbiology data was obtained from January 2010 to October 2014. Urinary isolates were identified using the Siemens MicroScan^®^ kits. Patients were included if they were 18 years of age or older, admitted between January 1, 2010 and October 1, 2014 with a urinary isolate positive for enterobacteriaceae, limited to *E. coli*, *K. pneumoniae*, and *P. mirabilis.* Only the first isolate of a certain bacterium per patient during the specified year was utilized in the analysis. Patients with repeated clinical isolates or polymicrobial urine cultures were also excluded.

A Siemens MicroScan^®^ query report was generated for urinary isolates of the specified pathogens during the time frame. Patients were included if the bacteria isolated was treated as clinically significant UTI. Urinalysis was not included in the patient selection due the retrospective nature of the study. Data collected included demographic information, past medical history, type of UTI, date and type of urine sample, organism isolated, susceptibility profile, empiric antibacterial agent(s) chosen for treatment, and intended duration of therapy. Infections were categorized as uncomplicated or complicated according to the Infectious Diseases Society of America (IDSA) consensus definitions [9].

Determinations of antibiotic susceptibilities for enterobacteriaceae were carried out by an overnight microdilution method with commercial dehydrated panels provided by Siemens MicroScan^®^ (Negative Urine Combo Panel Type 62). Breakpoints for the following drugs were also reported: amikacin, ampicillin/sulbactam, ampicillin, aztreonam, cefazolin, cefoxitin, ceftazidime, ceftriaxone, cefuroxime, ciprofloxacin, gentamicin, levofloxacin, nitrofurantoin, tobramycin, and trimethoprim/sulfamethoxazole [10].

The primary outcome was assessed by evaluating the change in susceptibility patterns for enterobacteriaceae (*E. coli*, *K. pneumoniae*, and *P. mirabilis*) isolated from urine cultures. We compared susceptibility rates before and after implementation of the updated CLSI breakpoints for cefazolin. MICs reported by the microbiology laboratory were based on the 2010 CLSI breakpoints, at the time the research was conducted. The MICs were then extrapolated to determine susceptibility based breakpoints published in the 2014 update. The secondary outcome was measured by comparing the total drug costs associated with the UTI for the hospital admission with that of cefazolin versus levofloxacin which was the most common agent used for complicated UTIs at our facility. Pricing for cefazolin was determined based on our contract through a Group Purchasing Organization (GPO).

Statistical analysis was completed using SAS v9.3 (SAS Institute, Inc., Cary, NC). Statistical significance for the primary outcome was assessed by implementing the two-proportion test of hypothesis, which was set at a *p* value less than 0.05 (5% level of significance) with a 95% confidence interval.

## Results

### Baseline demographics

Of the 204 patients screened, 14 were excluded as a result of not meeting the age criteria. The majority of patients were elderly females (n = 154) [67.3 ± 21.2 years old] (Table 2). Patients had a complicated UTI (78.4%) with majority of them having a documented past medical history significant for type 2 diabetes mellitus (29.3%) or end-stage renal disease (6.8%). Approximately 29% of patients (n = 56) had a documented allergy to penicillins and/or cephalosporins.

**Table 2:** Baseline demographics

Female, n (%)	154 (80.6)
Age (year), mean ± SD	67.3 ± 21.2
Race, n (%)	
Black	79 (41.4)
White	110 (57.6)
UTI classification, n (%)	
Complicated	149 (78.4)
Uncomplicated	41 (21.6)
Type 2 diabetes mellitus, n (%)	56 (29.3)
ESRD, n (%)	13 (6.8)
NH residents, n (%)	31 (16.2)
Penicillin/cephalosporin allergy, n (%)	56 (29.3)
ESBL-producing, n (%)	13 (6.5)

*ESRD* end stage renal disease, *NH* nursing home, *ESBL* extended-spectrum β-lactamase

### Microbiology data

*Escherichia coli* was the most common organism isolated (63.7%), followed by *K. pneumoniae* (22.1%) and *P. mirabilis* (14.2%). This frequency distribution reflected the historical pattern seen in previous years.

### Primary outcome

**Fig. 1:**
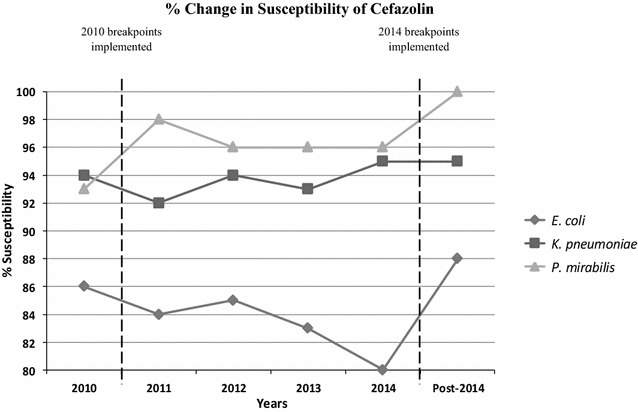
The change in percentages of susceptibility patterns for *E. coli*, *K. pneumoniae*, and *P. mirabilis* to cefazolin with CLSI breakpoint update

Prior to the 2014 CLSI cefazolin urine-specific breakpoints, a total of 163 isolates (85.8%) were susceptible to cefazolin, 7 (3.7%) were intermediate, and 20 (10.5%) were determined to be resistant (Fig. 1). After implementing the updated breakpoint of ≤16 mcg/mL (susceptible), the following changes were noted: 166 (87.4%) were susceptible, none were intermediate, and 24 (12.6%) were resistant. There was a non-statistically significant increase in the susceptibility rates for *E*. *coli* and *P*. *mirabilis*, from 80 to 88% (p = 0.077) and from 96 to 100% (p = 0.313), respectively. The susceptibility rate for *K*. *pneumoniae* was unchanged at 95% (p = 1.00). Susceptibility rates for ceftriaxone were 95% for *K*. *pneumoniae*, 91% for *E*. *coli*, and 100% for *P*. *mirabilis*. Similar rates were also observed for ceftazidime (95, 93, and 100%, respectively).

### Secondary outcome

Intravenous levofloxacin was the most common agent used for both empiric and definitive treatment. Patients received approximately 6 doses of levofloxacin intravenously during the inpatient visit. The direct medication cost of levofloxacin compared to cefazolin was based on the average duration of inpatient therapy. Total acquisition cost of intravenous levofloxacin was $20.04 whereas cefazolin was $8.82. An additional 33 isolates would be expected to be cefazolin susceptible based on the updated breakpoints, which could represent a reduction in drug acquisition cost by approximately $370.

## Discussion

Globally, FQs are frequently used as the first-line therapy for uncomplicated and often complicated UTIs [11, 12]. Concomitantly, there is rising antimicrobial resistance and increasing awareness of their potential side effects, toxicities, and their frequent association with *Clostridium difficile* infection. FQ resistance rates continue to increase both locally and nationally especially to *E. coli* where our susceptibility has decreased by 11.3% in the last 2 years. In this context, the use of β-lactams offers many advantages, especially in patients who are not candidates for trimethoprim/sulfamethoxazole therapy. In particular, the use of cefazolin remains as an affordable and effective antimicrobial that may potentially be considered in many settings as a first-line therapy for the treatment of UTIs. Our study provides further evidence to support the use of cefazolin as a first-line therapy in the management of UTIs in some settings even after implementing the new urine-specific breakpoints among *E. coli* and *P. mirabilis* isolates. We did not observe any statistical difference for any of the organisms when comparing the 2010–2014 and post-2014 results. Indeed, compared to baseline, in vitro susceptibility rates remain high despite implementing the new urine specific breakpoint. In addition to preventing potential side effects and toxicities associated with the use of FQs, the substitution of cefazolin for treatment of both complicated and uncomplicated UTIs may decrease drug costs by approximately 60% [11]. Finally, after implementation of the updated cefazolin breakpoints, susceptibility rates were comparable between first and third generation cephalosporins.

Current UTI and pyelonephritis guidelines, published in 2011, do not recommend cefazolin as a first-line choice for treatment of UTIs [9]. Recently, there has been renewed interest in repurposing older or narrower-spectrum antibiotics due to increasing rates of resistance and paucity of novel agents [3, 7, 12, 13]. Greater knowledge of antimicrobial PK, PD, and resistance mechanisms will allow modifications to susceptibility breakpoints. From an antimicrobial stewardship perspective, evaluation and use of cefazolin is potentially of great interest. Data for cefazolin use in UTIs remains limited despite increasing use at some facilities. A recent study however demonstrated noninferiority of cefazolin compared to ceftriaxone for acute pyelonephritis [12]. High rates of susceptibility to cefazolin, comparable to those observed with third generation cephalosporins, as presented by our data, suggest that empirical use of cefazolin may be warranted. Avoiding widespread use of third generation cephalosporins for UTIs in patients requiring hospitalization may limit development of resistance. Third generation cephalosporins have been associated with subsequent infections caused by vancomycin resistant enterococci (VRE), extended spectrum β-lactamase (ESBL) producing *K*. *pneumoniae*, and *C*. *difficile* [11].

The ability to transition patients from cefazolin to oral cephalosporins to complete therapy represents an area of uncertainty [13]. A recent in vitro study was conducted in an attempt to determine whether cefazolin could be used as a surrogate marker for cefpodoxime for urinary tract isolates. The authors found significantly higher categorical agreement with cefazolin compared to cefuroxime. Cefuroxime was noted to have better major and very major error rates than cefazolin. This data may allow clinicians to convert patients to an oral cephalosporin much sooner, potentially decreasing length of stay related to UTI diagnoses. For patients with an uncomplicated UTI who require treatment in an outpatient setting, cefazolin urine-specific MICs may be used as a surrogate for oral cephalosporins such as cephalexin and cefpodoxime. Although efficacious, FQs are not benign, as they were once considered. The Food and Drug Administration (FDA) recently strengthened warnings on FQs urging clinicians to avoid their use in uncomplicated UTIs due to serious toxicities and potentially irreversible adverse events [14]. Avoidance of FQs in patients with uncomplicated UTIs may be achieved with increased use of oral cephalosporins in the outpatient setting.

There are several limitations to this study. The small sample size from a single center institution limits its external validity. In addition, our cost analysis did not account for indirect cost (such as nursing time, insertion and maintenance of an intravenous line, or frequency of cefazolin administration). Furthermore, the cost analysis did not account for patients with a penicillin allergy, which would likely decrease the usage of cefazolin in this population.

## Conclusion

The institution of newer urinary breakpoints along with increasing antimicrobial resistance to first-line UTI agents such as FQs or trimethoprim/sulfamethoxazole may require instead the use of β-lactams for treating UTIs. Third-generation cephalosporins should be avoided if possible due to their frequent association with VRE, ESBL producing *K*. *pneumoniae*, and *C*. *difficile*. The use of cefazolin and potentially other first-generation cephalosporins offers an affordable, safe, and efficacious antimicrobial alternative as a first-line therapy in the management of complicated and uncomplicated UTIs. Further research is needed to ascertain in vitro effect as well as clinical effect in settings that may be significantly affected by the updated urine-specific breakpoints.

## Abbreviations

UTI: urinary tract infections
MIC: minimum inhibitory concentrations
CLSI: Clinical and Laboratory Standards Institute
PK: pharmacokinetic
PD: pharmacodynamics
FQs: fluoroquinolones
AST: antibiotic susceptibility testing
IDSA: Infectious Diseases Society of America
VRE: vancomycin resistant enterococci
ESBL: extended-spectrum β-lactamase
FDA: Food and Drug Administration
ESRD: end stage renal disease
NH: nursing home
